# The Story of the Insane from Year to Year

**Published:** 1902-09-20

**Authors:** 


### THE STORY OF THE INSANE FROM YEAR TO YEAR.

Fifteenth Annual Series.

### DORSET COUNTY ASYLUM.

On January 1st this Asylum contained 718 patients, and on December 31st the number had risen to 736. There were 119 first admissions, 26 readmissions, 50 discharged recovered, 7 discharged relieved, 23 discharged not relieved, and 47 died. The average daily number resident was 732, and the total number under care was 845. Excluding tranfers the recoveries stand at the creditable percentage of 43·10 on the admissions, and the deaths at the low percentage of 6·42 on the average number resident. Of the year's deaths 18 were caused by cerebral and spinal diseases, 2 to consumption, and 5 to other lung diseases. Of the admissions, 27 owed their insanity to moral causes, and 112 to physical causes. Among the former, mental anxiety was the most potent, and among the latter, hereditary influence, as usual, was the greatest factor, intemperance in drink accounted for only 4. When speaking of the nature of the cases admitted, Dr. MacDonald says that 70 per cent. of the men were classed as incurables, and that the women were even less favourable than the men. Some medical superintendents clamour for the erection of acute hospitals wherein recent cases could be treated, and these men fancy that their percentage of recoveries would be greatly increased if such hospitals were at hand. We have always doubted that such would be the case, and we should like to have Dr. MacDonald's opinion on the subject as he is well known as one of the ablest superintendents in England. It is doubtful whether any journal has so often pointed out the evil effects of unsuitable marriages in the propagation of insanity as The Hospital, and we are glad to quote the following sentence from this report: "The cases thus classified exemplify the baneful results of errors committed by the too frequent disregard of the most ordinary laws of consanguinity. Advice is of no avail—far otherwise —for often when sought it is not followed, and the reckless mental blindness so constantly exhibited, causes the thoughtful onlooker to stand aghast in utter amazement." And further on we come upon the following words:— "When the awful consequences of ancestral risk have established a firm footing in the public mind there may be hope for the future; but that each year is revealing more painfully and more truthfully the scourge of heredity in relation to mental disease needs no confirmation." We fear the day is far distant when the public will pay attention to such warnings as Dr. MacDonald gives, but that is no reason why he should not again and again express his views. The Committee of Visitors say that "the success which has attended the administration of the asylum is mainly due to the untiring supervision taken in the welfare of the institution by the energetic superintendent, Dr. MacDonald." The weekly rate of maintenance is 9s. 4d. per head.

### THE MONMOUTHSHIRE ASYLUM AT ABERGAVENNY.

At the beginning of the year this asylum contained 1,065 patients, and at the end of the year 1,098 were in residence. There were 193 first admissions, 24 readmissions, 71 discharged recovered, 21 discharged relieved, and 89 deaths. The average number resident was 1,078, and the total number under treatment was 1,278. The recoveries stand at 33·7 per cent of the admissions, and the deaths at 8·2 per cent. of the average number resident. Of the deaths 35 were due to cerebral and spinal diseases, 7 to consumption, 9 to pneumonia, 4 to other lung diseases, and 2 to ulceration of the intestine. Of the admissions moral causes account for the insanity in 15 instances, and physical causes in 167. Domestic trouble heads the list among the former with 9 cases, and among the latter hereditary influence accounts for 34 and intemperance in drink for 22. It is well known that nothing is so potent in increasing the recovery rate as early asylum treatment, and that nothing militates so strongly against recovery in curable cases as delayed treatment. Dr. Glendinning shows this conclusively enough in his experience during the past year. Of 87 patients whose insanity dated less than three months, 39, or nearly 45 per cent., recovered, while of 57 patients whose insanity had lasted a year before they were sent to the asylum, only 7 per cent. recovered. Among the deaths was one from diphtheria; and there was also under treatment a mild case of scarlatina. With these two exceptions the asylum was free from infectious disease. Drs. Nelis and Black, the assistant medical officers, interest themselves greatly in training the nurses and attendants, and seven of the staff obtained the nursing certificate. In this asylum the admirable plan is followed of presenting the successful candidates with medals and increasing their wages. The committee testify to their "high appreciation of the zealous services rendered by Dr. Glendinning and his able assistants." The weekly rate of maintenance is 8s. 2d. per head.

### THE LEICESTER BOROUGH ASYLUM.

On January 1st there were 565 patients in residence, and at the end of the year the numbers had risen to 797; but this large increase was owing to the transfer of cases from other asylums to fill up the accommodation provided by the extensions opened during the year. There were 347 first admissions, 61 readmissions, 75 discharged recovered, 34 discharged not improved, 12 discharged relieved, and 54 deaths. The average number resident was 722. Excluding the transfers, the recoveries work out at 40·7 per cent., and the deaths at 7·4 per cent. on the average number resident. Of the year's deaths 28 were caused by cerebral and spinal diseases, 5 by consumption, and 9 by other lung diseases. Various moral causes, such as domestic trouble, adverse circumstances, and religion account for the insanity in 27 of the admissions, and among the physical causes intemperance in drink is credited with being the cause in 24 instances, hereditary influence in 35, and previous attacks in 28. During the last decade, the population of the borough has increased from 177,000 to 214,443, and the insane in the asylum from 387 to 590. The weekly rate of maintenance was 10s. 8d. per head.

### HANTS COUNTY ASYLUM AT FAREHAM.

Here there was increase during the year of 41 patients and when the year ended there were 1,138 in residence. There were 220 first admissions, 40 readmissions, 72 discharged recovered, 11 discharged relieved, and 114 deaths. The average number resident was 1,122, and the total number under treatment was 1,357. The percentage of recoveries on the admissions stands at 27·7, which is lower than the County Asylum average, and also lower than the Hants average for many years. The deaths stand at 11·2 per cent on the average number resident. Of the year's admission 33 were supposed to owe their insanity to such moral caus[illegible] as domestic trouble and religious excitement. Among [illegible] physical causes, intemperance in drink accounts for [illegible]

hereditary influence for 71, previous attacks for 57, and sunstroke for 2. The asylum physician has even a more difficult task in the diagnosis of disease than his professional brother. The usual symptoms are often absent in the insane, or what is worse, they are misleading. Dr. Worthington has had an outbreak of typhoid fever, and two of the cases illustrate this peculiarity in a very striking manner. "An epileptic idiot of very low type, died in a fit. A post-mortem examination showed that a typhoid ulcer had ruptured, and was the cause of death, He had no symptoms of fever during life, and was up and took his food well the day he died. In another case at the post-mortem examination it was ascertained that the patient's heart was greatly affected, and that she had three large typhoid ulcers. She had not a single symptom of typhoid during life." What makes the latter case more remarkable is that she had been in the infirmary ward for 18 months, and that the nurse in charge had two years' experience in a fever hospital. The committee say they are well satisfied with the general management of the asylum. The weekly rate of maintenance is 9s. 4d. a head.

